# Identification of downstream metastasis-associated target genes regulated by LSD1 in colon cancer cells

**DOI:** 10.18632/oncotarget.14778

**Published:** 2017-01-21

**Authors:** Jiang Chen, Jie Ding, Ziwei Wang, Jian Zhu, Xuejian Wang, Jiyi Du

**Affiliations:** ^1^ Department of Gastrointestinal Surgery, The First Affiliated Hospital of Chongqing Medical University, Chongqing, China; ^2^ Department of Gastrointestinal Surgery, Guizhou Provincial People's Hospital, Guiyang, China; ^3^ Department of Mini-invasive Surgery, Guiyang Hospital of Guizhou Aviation Industry Group, Guiyang, China; ^4^ Department of Gastrointestinal Surgery, The First People's Hospital of Guiyang, Guiyang, China

**Keywords:** colon cancer, LSD1, gene expression profiling, target gene, epigenomics

## Abstract

**Purpose:**

This study aims to identify downstream target genes regulated by lysine-specific demethylase 1 (LSD1) in colon cancer cells and investigate the molecular mechanisms of LSD1 influencing invasion and metastasis of colon cancer.

**Method:**

We obtained the expression changes of downstream target genes regulated by small-interfering RNA-LSD1 and LSD1-overexpression via gene expression profiling in two human colon cancer cell lines. An Affymetrix Human Transcriptome Array 2.0 was used to identify differentially expressed genes (DEGs). We screened out LSD1-target gene associated with proliferation, metastasis, and invasion from DEGs via Gene Ontology and Pathway Studio. Subsequently, four key genes (CABYR, FOXF2, TLE4, and CDH1) were computationally predicted as metastasis-related LSD1-target genes. ChIp-PCR was applied after RT-PCR and Western blot validations to detect the occupancy of LSD1-target gene promoter-bound LSD1.

**Result:**

A total of 3633 DEGs were significantly upregulated, and 4642 DEGs were downregulated in LSD1-silenced SW620 cells. A total of 4047 DEGs and 4240 DEGs were upregulated and downregulated in LSD1-overexpressed HT-29 cells, respectively. RT-PCR and Western blot validated the microarray analysis results. ChIP assay results demonstrated that LSD1 might be negative regulators for target genes CABYR and CDH1. The expression level of LSD1 is negatively correlated with mono- and dimethylation of histone H3 lysine4(H3K4) at LSD1- target gene promoter region. No significant mono-methylation and dimethylation of H3 lysine9 methylation was detected at the promoter region of CABYR and CDH1.

**Conclusion:**

LSD1- depletion contributed to the upregulation of CABYR and CDH1 through enhancing the dimethylation of H3K4 at the LSD1-target genes promoter. LSD1- overexpression mediated the downregulation of CABYR and CDH1expression through decreasing the mono- and dimethylation of H3K4 at LSD1-target gene promoter in colon cancer cells. CABYR and CDH1 might be potential LSD1-target genes in colon carcinogenesis.

## INTRODUCTION

Colon cancer is known as the third most common malignancy throughout the world [1]. Recurrence and metastasis are the primary causes of death among colon cancer patients. Growing evidence has indicated that lysine-specific demethylase 1 is associated with tumorigenesis and growth, invasive ability, metastasis, and therapeutic resistance [2–5]. A broad scope of genetic and epigenetic modifications plays an important role in the development and tumorigenesis of colon cancers. The epigenetic changes are related to DNA methylation and histone modification [6, 7].

LSD1 is regarded as the first discovered histone demethylase, which is required in Snail/ Slug-mediated transcriptional repression during epithelial mesenchymal transition (EMT); in short supply of LSD1, Snail/Slug is unable to repress CDH-1 transcription [8–10]. Used as a molecular “hook” that affects with LSD1- CoREST complex mutually, the Snail/Gfi-1 domain of Snail/Slug ensembles a histone H3-like structure; this complex is conveyed to its targeted gene promoters through the binding of E-box and zinc-finger motifs [7–9]. The LSD1-CoREST complex works as a reversible nanoscale binding clamp and recruits and anchors various substrate peptides with high sequence similarity to H3-histone tail [10–13].

LSD1 belongings to a flavin-dependent amine oxidase family. Thus, LSD1 catalyzes the demethylation of mono- methylated and di-methylated histone H3 lysine 4 (H3K4) and H3lysine 9 (H3K9) through the redox process. LSD1 overexpression facilitates proliferation, migration, and invasion of various malignancies [5, 14, 7], such as acute myeloid leukemia [15], Ewing sarcoma [16], breast cancer [17], bladder carcinoma, small cell lung cancer, and colorectal carcinomas [6]. LSD1 overexpression indicates that LSD1 inhibitors may provide important therapeutic benefit in substantial tumors [18–21]. Knockdown of LSD1 with small-interfering RNAs (siRNAs) makes for the suppression of proliferation and metastasis of various cancer cells [22–25].

Our previous studies showed that Silencing LSD1 gene damages proliferation and invasiveness, and stimulates apoptosis of colon cancer cells *in vitro* [3]. We speculated that LSD1 can downregulate the H3K4 and H3K9 methylation levels of target gene promoter regions through the activity of histone-specific-demethylase and regulation of the transcription and translation of target genes via the epigenetic modification pathway. Such downregulation affects the invasion and metastasis of colon cancer.

Therefore, we attempted to investigate deeply the mechanisms of invasion and metastasis involving LSD1-target genes in colon cancer cells. We performed gene expression profiling and ChIp assay to identify the LSD1-target gene transcriptional regulation in colon cancer cells. Our findings displayed that LSD1-target genes, namely, CABYR and CDH1 might be potential LSD1-target genes in colon cancer cells, and revealed the underlying mechanisms of invasion and metastasis in colon cancer.

## RESULTS

### Differential gene expression changes in SW620 and HT29 cells

**Figure 1:**
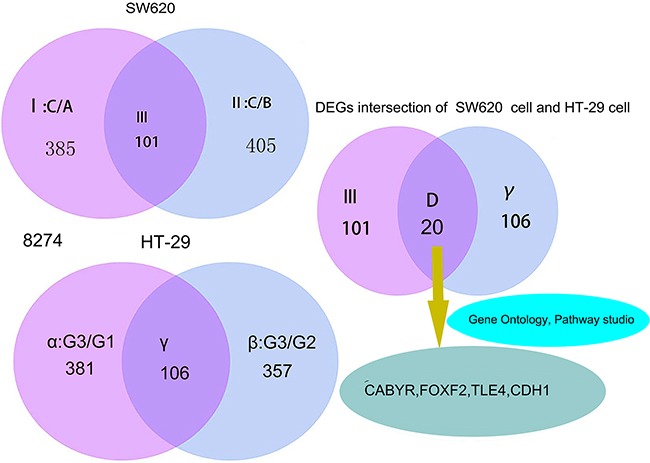
Gene screening process and DEGs intersection of SW620 and HT-29 cells I: DEGs set selected from 8274 DEGs of SW620 cell for log2|Fold change| ≥ 2 II: DEGs set selected from 8274 DEGs of SW620 cell for log2|Fold change| ≥ 2 III: The intersection of I and II α: DEGs set selected from 8274 DEGs of HT-29 cell for log2|Fold change| ≥ 2 β:DEGs set selected from 8274 DEGs of HT-29 cell for log2|Fold change| ≥ 2 γ: The intersection of α and β D: The intersection of III and γ DEGs intersection of SW620 cell and HT-29, means the number of DEGs intersection of screening for SW620 cell and HT-29 cell via gene expression profiling. According to differently treated factors, the following were determined: SW620 cell of C group treated by LSDI-siRNA;HT-29 cell of G3 group treated by LSD1-overexpressionThe DEGs set of D must meet the following conditions:The expression of the DEGs increased in III and simultaneously decreased in γ (and vice versa).

**Figure 2:**
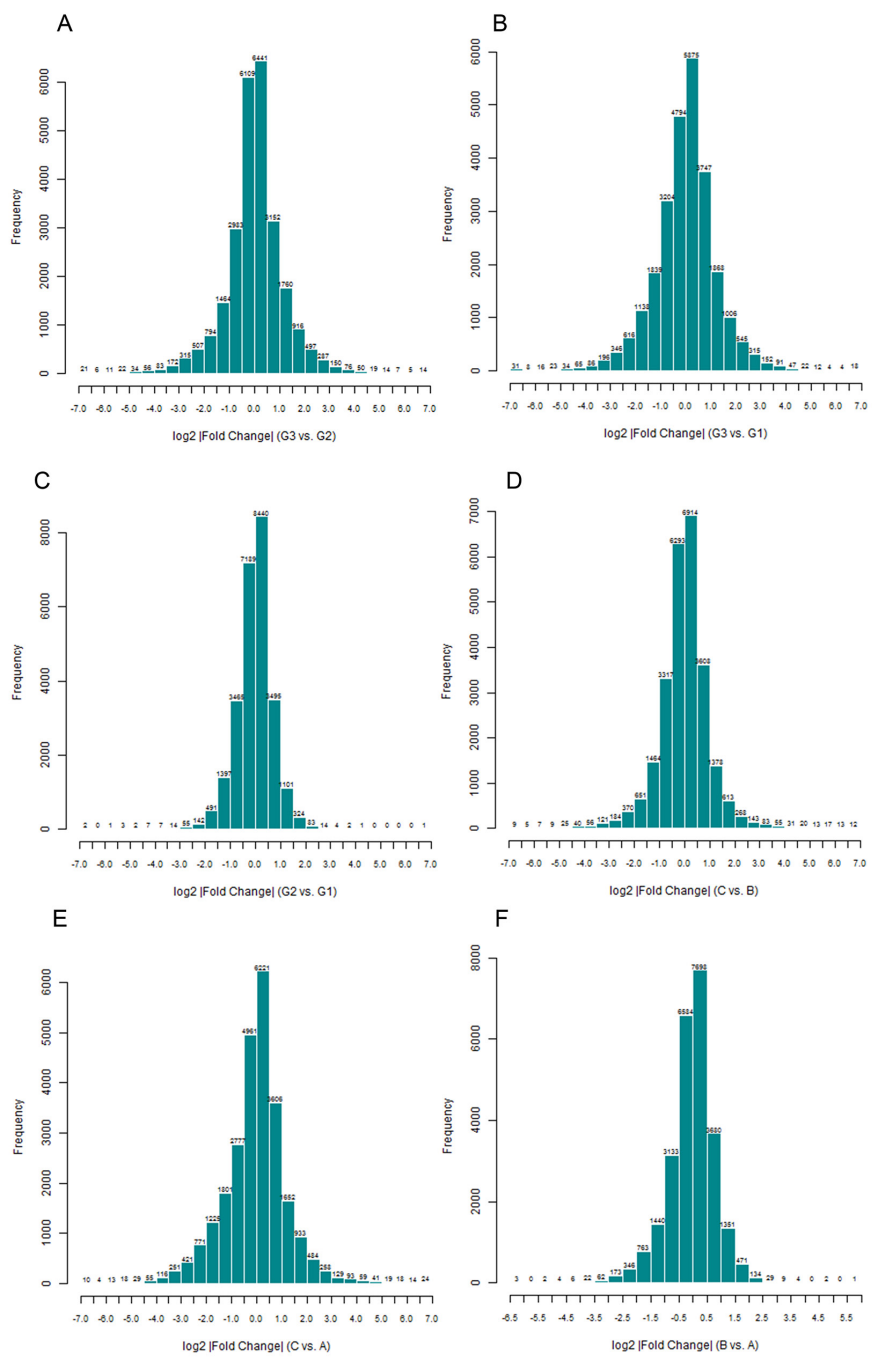
Normal distribution histogram analysis of differentially expressed genes(DEGs) The result suggested the distribution of signal difference of all gene probes detected in grouping comparison. The fold change is calculated by using Rosetta Resolver 7.2 software error model. The comparison among groups is performed using Amersham Pairwise Ration Builder software. X axis: the fold change expressed by log2. Y axis: amount of probes.

**Figure 3:**
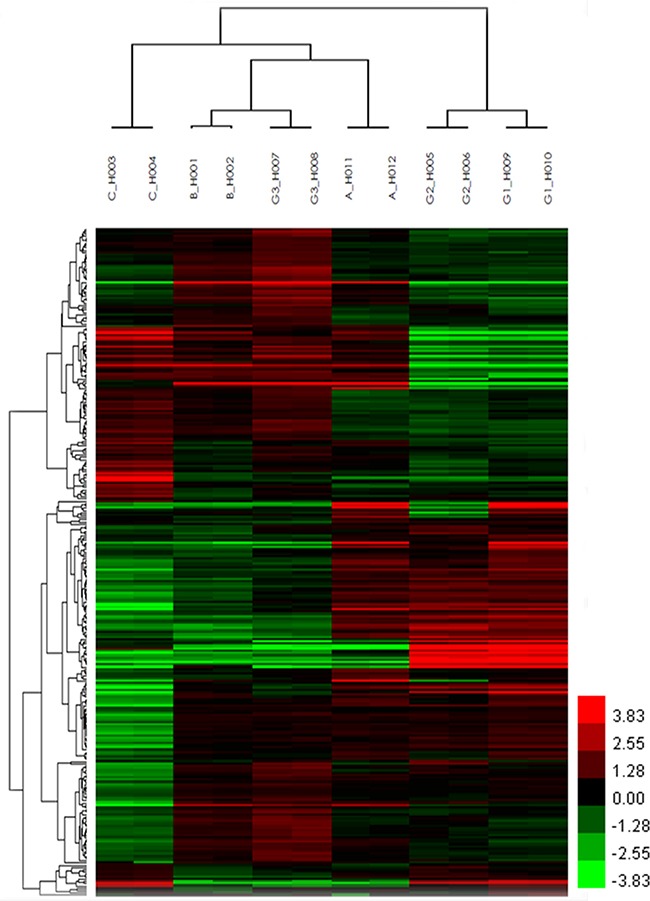
Unsupervised hierarchical clustering analysis of DEGs Unsupervised hierarchical cluster image shows the differential gene expression profiles in A, B, C, G1, G2, and G3 groups. The Heat maps display a color scale: red indicates upregulation, whereas green represents downregulation. The columns and rows in the heat maps represent genes and microchips, respectively. Color brightness represents the degree of difference, as shown in the color bar.

**Figure 4:**
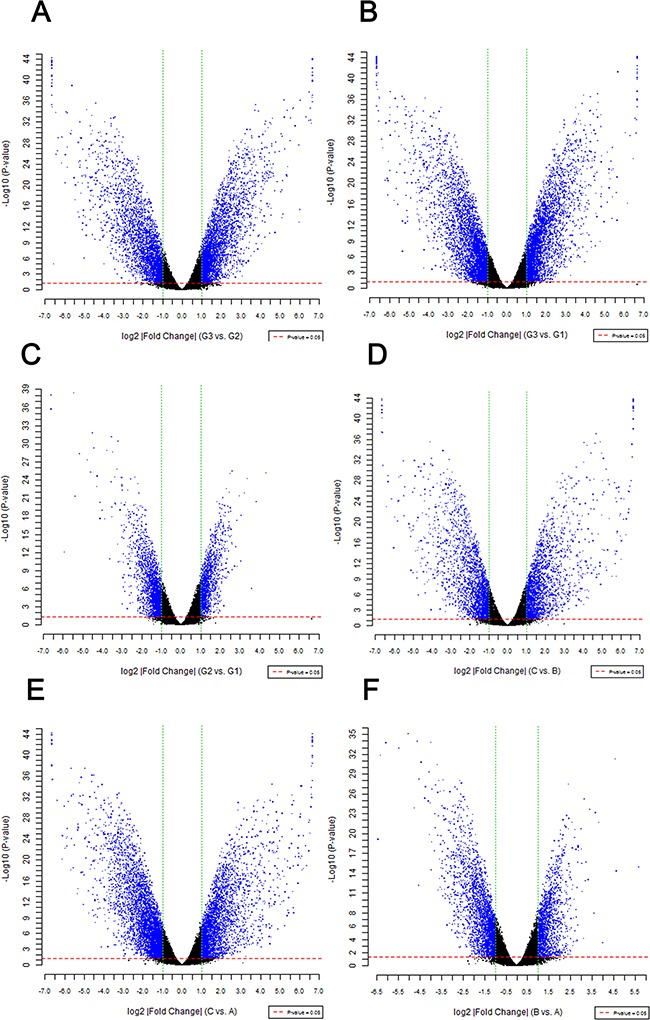
Difference significance analysis of DEGs The volcano plot shows the distribution of DEGs in grouping comparison and fold change on x-axis. The P value on y-axis represents the significance of the difference. Red and green dotted lines represent the threshold value of P and multiple screening, respectively. Each point in the diagram is a detected gene probe. Blue dots represent the difference probes above each group. |Fold change|≧1 and P-value<0.05. The significance of gene differential expression between experimental and control groups is indicated.

**Figure 5:**
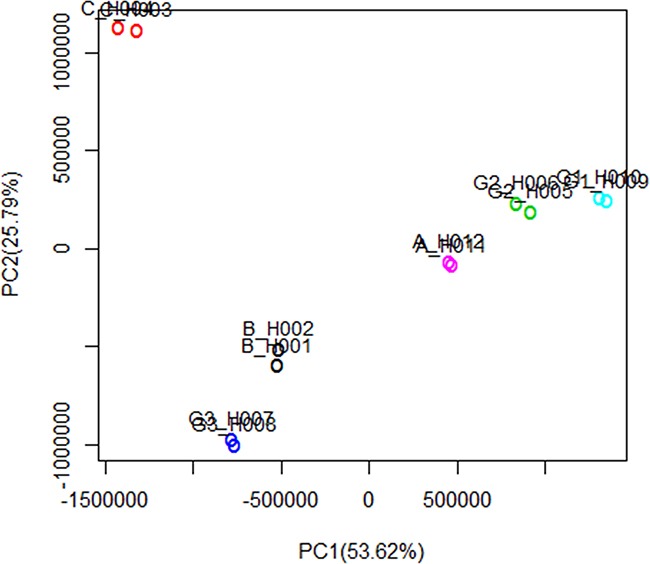
Principal component analysis of DEGs Horizontal axis represents the first principal component scores of gene expression profiles, and vertical axis represents the second principal component scores. The first and second principal components explain 53.62% and 25.79% of the variance, respectively. The interpretation degree of cumulative variance is 79.41. The first two principal components of gene expression profiles illustrate the Euclidian separation of individual microarrays. The experimental data show good repeatability. The different experimental groups are separated from one another, thereby showing good specificity.

A total of 8274 DEGs were identified according to the expression ratio of genes(the expression ratio= log2|Fold change|C vs. A; log2|Fold change|C vs. B; log2|Fold change|G3 vs. G1; log2|Fold change|G3 vs. G2, log2|Fold change| ≥ 1 and P <0.05, Figure 1). Among these genes, 3633 were upregulated, and 4642 were downregulated in the C group compared with the control group(A and B groups). In G3 group, 4047 were upregulated, and 4240 were downregulated (Table 1) compared with the control group (G1 and G2 groups). Histogram analysis showed the normal distribution of DEGs in gene probes and indicated that upregulated and downregulated genes were approximately equivalent in Groups C and G3 (Figure 2). We selected the 290 expressed gene probes for hierarchical clustering analysis. Our correlation analysis revealed that the grouping was reasonable, and data could be directly applied to further analysis (Figure 3). The volcano plot showed the distribution of DEGs in grouping comparison and indicated a significant difference between experimental and control groups (Figure 4). Principal component analysis of DEGs demonstrated that different experimental groups were separated from one another and showed good specificity (Figure 5).

**Table 1:** DEGs

number	Comparison group	up-regulated amount	down-regulatedamount
**1**	B/A	1817	2754
**2**	C/A	3633	4642
**3**	C/B	2505	2711
**4**	G2/G1	1531	2061
**5**	G3/G1	4047	4240
**6**	G3/G2	3757	3376

### Selection of LSD1-target genes for *in vitro* screening

RNA-Seq was performed to analyze the DEGs of SW620 and HT-29 cells treated with LSD1-siRNA and LSD1-overexpression, respectively, and determine the function and pathway of each LSD1-target gene. Genes with a significant change of twofold change or more were screened. We found 8274 DEGs, among these gene,770 were expressed in C/A(I) of SW620 cells, and 810 were expressed in C/B(II) of SW620. A total of 101 genes were significantly differentially expressed and common to both C/A(I) and C/B(II). Among these DEGs,381 were expressed in G3/G1(α) of HT-29 cells, 357 were expressed in G3/G2(β) of HT-29, and 106 DEGs representing γ intersection showed overlapped expression between G3/G1(α) and G3/G2(β). Statistical analysis(P <0.01) allowed the screening out of 20 genes that exhibited highly significant differences according to the following criteria: log2|Fold change| ≥ 2. DEGs expression increased in III and simultaneously decreased in γ, whereas the expression of DEGs reduced in III and simultaneously increased in γ (Figure 1 and Table 2).

**Table 2:** 20 DEGs of intersection of SW620 cell and HT-29 cell (the intersection of III and γ), expressed as the ratio of experimental group and control group

GeneSymbo	Description	Genbank Accession	Fold ChangeC/A	Fold ChangeC/B	Fold ChangeG3/G1	Fold ChangeG3/G2
CABYR	Calcium Binding Tyrosine-(Y)-Phosphorylation Regulated	NM_153770	5.385	5.008	−3.183	−2.065
FOXF2	d ForkheadBox F2, eficiency promotes epithelial-mesenchymal transition and metastasis of basal-like breast cancer	NM_001452	4.674	3.582	−5.058	−5.140
NELL2	Neural EGFL Like 2	NM_001145108	4.748	5.247	−4.442	−4.448
TLE4	Transducin-Like Enhancer Of Split 4	NM_001282748	5.021	4.517	−4.234	−4.185
CDH1	Cadherin 1	NM_004360	6.364	5.536	−5.189	−4.985
COPZ2	Coatomer Protein Complex Subunit Zeta 2	NM_016429	4.199	4.404	−5.323	−5.083
SLC9A2	Solute Carrier Family 9 Member A	NM_003048	4.253	4.155	−4.115	−4.051
STAP-2	signal transducing adaptor family member 2	NM_145934	4.068	3.965	−4.638	−4.687
ADGRF1	Adhesion G Protein-C oupled Receptor F1	NM_153840	−6.644	−6.644	6.594	5.389
BDNF	Brain-Derived Neurotrophic Facto	NM_001143805	−2.207	−2.861	4.163	4.542
CD40	CD40 Molecule, TNF Receptor Superfamily Member 5	NM_001250	−5.190	−5.509	4.148	5.354
EREG	Epiregulin	NM_001432	−5.885	−3.778	2.729	3.462
S100A14	S100 Calcium Binding Protein A14	NM_020672	−6.600	−6.644	2.309	2.141
VAV1	Vav 1 Guanine Nucleotide Exchange Factor	NM_001258206	−2.564	−2.687	2.579	2.009
AKR1B1	aldo-keto reductase family 1 member B	NM_001628	−5.135	−4.741	6.643	6.643
CENPA	centromere protein A	NM_001809	−3.836	−3.259	4.056	4.091
F2R	coagulation factor II thrombin receptor	NM_001992	−5.797	−3.639	4.760	4.123
NFIB	nuclear factor I B	NM_005596	−4.490	−5.771	4.791	5.244
NR4A2	nuclear receptor subfamily 4, group A, member 2	NM_006186	−6.644	−6.644	3.706	3.844
MAGEA6|MAGEA3	MAGE family member A6/A3	NM_005362	−6.644	−6.644	6.644	6.644

(log2|Fold change| ≥ 2, p <0.01)

Subsequently, we screened out LSD1-target gene associated with proliferation, metastasis, and invasion from set of D via bioinformatics technology and literature search (Figure 1 and Table 10). Although numerous genes display multiple functions, this study focused on the major pathway and function of the LSD1-target gene. which played a major role in proliferation, metastasis, and invasion. We further focused on four key genes (CABYR, FOXF2, TLE4, and CDH1 ) related to proliferation, apoptosis, tumorigenesis, invasion and metastasis of cancer cells (Table 3).

**Table 10:** Sequences of primers used forRT-PCR

Gene	Forward primerReverse primer	Product Size (bp)
Homo-CABYR-FHomo-CABYR-R	5-AACCAGCCACCCCTAAGACT-35-CTTCAGCAGCCTCTGAGCTT-3	222
Homo-FOXF2-FHomo-FOXF2-R	5-ACTCAGGTGGGAAGATGTGC-35-TTCAGATTGGGGAACGCTAC-3	203
Homo-NELL2-FHomo-NELL2-R	5-AAGTAGTGGCCATCGGAATG-35-GCCTAGAGGCAAGTCTGTGG-3	208
Homo-TLE4-FHomo-TLE4-R	5-GGATCTGCACAACCAGACCT-35-CTCTCCAGTTGGGCAGTAGC-3	208
Homo-ADGRF1-FHomo-ADGRF1-R	5-GGCCCAGTCGAAGAATATCA-35-GGTAGCAGTTCTGGGGATCA-3	244
Homo-BDNF-FHomo-BDNF-R	5- AAACATCCGAGGACAAGGTG-35-AGAAGAGGAGGCTCCAAAGG-3	209
Homo-CD40-FHomo-CD40-R	5-GCAGGCACAAACAAGACTGA-35- TCGTCGGGAAAATTGATCTC-3	194
Homo-EREG-FHomo-EREG-R	5- TGCTCTCAGCTGATGTGTCC-35- ATGTGGCCTTGGTTGAAGAC-3	187
Homo-S100A14-FHomo-S100A14-R	5- CTTCTGAGCTACGGGACCTG-35- CCAGAGGGAGTTCTCAGTGC-3	201
Homo-VAV1-FHomo-VAV1-R	5- TGGTGTCCTTCTGTGTCAGC-35- CTTGAGGCCGAACTTCTCAC-3	151
Homo-β-actin-FHomo-β-actin-R	5- CATTAAGGAGAAGCTGTGCT-35- GTTGAAGGTAGTTTCGTGGA-3	208

**Table 3:** Differential expression genes involved in cell proliferation, apoptosis, tumorigenesis, invasion and metastasis of cancer cells

GeneSymbo	Functional annotation
CABYR	a calcium-binding tyrosine phosphorylation–regulated protein that was identified as a novel cancer testis antigen in lung cancer, a negative correlation existed between the expression level of CABYR-a/b and TRAIL-induced apoptosis in lung cancer cells.
FOXF2	a novel EMT-suppressing transcription factor in basal-like breast cancer (BLBC), FOXF2 deficiency enhances metastatic ability of BLBC cells by activating the EMT program through upregulating the transcription of TWIST1.
TLE4	TLE4 might be a valuable prognostic marker of CRC progression, some studies provide evidence for diverse molecular mechanism by which TLE4 can promote tumorigenesis of CRC.
CDH1(E-cadherin)	CDH1(E-cadherin) gene involved in invasion and metastasis of cancer cells, LSD1 regulates EMT via demethylation of CDH-1 gene.

**Table 4:** GO (molecular function) Enrichment analysis of top10 probe sets

Gene set name/MF	Genes in overlap	P Value
GO:0000166~nucleotide binding	**958**	2.76836920947265E-12
GO:0017076~purine nucleotide binding	**818**	2.27653759745962E-10
GO:0001882~nucleoside binding	**697**	4.13440394909436E-10
GO:0030554~adenyl nucleotide binding	**683**	4.56712529338218E-10
GO:0001883~purine nucleoside binding	**692**	5.21219972864165E-10
GO:0032553~ribonucleotide binding	**778**	3.04548998745947E-09
GO:0032555~purine ribonucleotide binding	**778**	3.04548998745947E-09
GO:0005524~ATP binding	**638**	3.34097494597093E-09
GO:0032559~adenyl ribonucleotide binding	**644**	5.90707148610908E-09
GO:0008134~transcription factor binding	**244**	8.03044993308827E-08

**Table 5:** GO (biological process)Enrichment_analysis of top10 probe sets

Gene set name/BF	Genes in overlap	P Value
GO:0007049~cell cycle	**365**	1.94110536461176E-10
GO:0000278~mitotic cell cycle	**189**	4.21691388274489E-09
GO:0022402~cell cycle process	**269**	1.51883084093203E-08
GO:0044265~cellular macromolecule catabolic process	**333**	3.19055642301562E-08
GO:0044257~cellular protein catabolic process	**283**	3.43439856100022E-08
GO:0051603~proteolysis involved in cellular protein catabolic process	**281**	4.91554222912098E-08
GO:0043632~modification-dependent macromolecule catabolic process	**270**	5.67201953812086E-08
GO:0019941~modification-dependent protein catabolic process	**270**	5.67201953812086E-08
GO:0008219~cell death	**329**	6.15463111005308E-08
GO:0030163~protein catabolic process	**289**	7.83970928349477E-08

**Table 6:** GO (cellular component) Enrichment_analysis of top10 probe sets

Gene set name/CC	Genes in overlap	P Value
GO:0031974~membrane-enclosed lumen	**878**	4.04829543975648E-34
GO:0070013~intracellular organelle lumen	**845**	1.76212953596665E-33
GO:0043233~organelle lumen	**859**	7.30324518381222E-33
GO:0031981~nuclear lumen	**692**	9.76447056288165E-28
GO:0005739~mitochondrion	**524**	5.1209357005266E-22
GO:0005829~cytosol	**614**	3.30538132505074E-20
GO:0043228~non-membrane-bounded organelle	**1083**	1.99568162323316E-17
GO:0043232~intracellular non-membrane-bounded organelle	**1083**	1.99568162323316E-17
GO:0005654~nucleoplasm	**419**	5.69987256898464E-16
GO:0031975~envelope	**309**	4.72684808018608E-15

### Gene ontology (GO) functional enrichment analysis of DEGs

**Figure 6:**
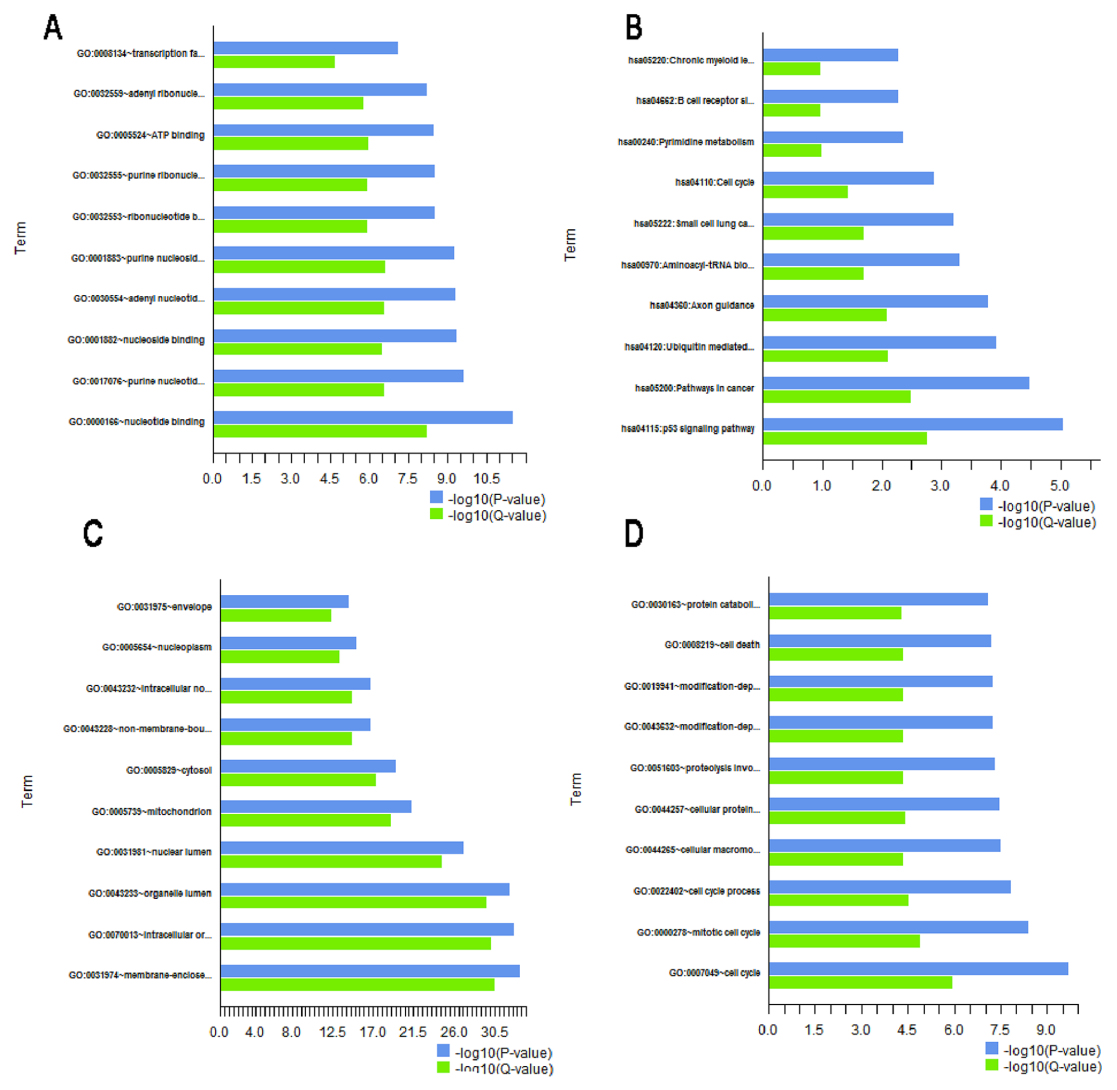
Gene annotation(GO) enrichment analysis for top 10 probe sets The bar plot shows the enrichment scores (−log10[P value, Q value]) of significant enrichment GO terms. The ontology covers four domains: **A**. pathway, **B**. molecular function, **C**. biological process, and **D**. cell component. (P≤0.05 and Q <0.05 are recommended). Enrichment of pathway analysis is performed with KEGG.

Biological effects of LSD1-target genes for top 10 probe sets of pathway and GO enrichment were proved by GO, pathway, cluster, and network analyses. GO analysis could distribute genes to various functional categories according to GO types. Three GO classifications which are called biological process [BP], molecular function [MF], and cell component [CC] were explored using DAVID. Pearson chi-square test was used to determine whether there was an overlap between the differently expressed list and GO annotation list. In this study, 2878 genes in the downstream target genes referred to biological processes; 7306 genes involved in cellular components, and 6930 genes have connection with molecular functions. The results suggested that 365 target genes involved in cell cycle accounted for the largest number in the BP. The target genes involved in cellular macromolecule catabolic process were 333, and 958 of the target genes involved in nucleotide binding were the majority in MF. Both the target genes involved in nonmembrane-bound organelle and those involved in intracellular nonmembrane-bound organelle were the majority in the CC. Both of these target genes were 1083, and they were involved in membrane-enclosed lumen in the CC (Tables 4–6, Figure 6). P-value indicates the significance of GO term enrichment in DEGs. The low P value resulted in considerably significant GO term (P ≤ 0.05, FDR-adjusted, Q-value <0.05 was recommended).

### KEGG pathway analysis

Pathway analysis helps to comprehend the further biological functions of genes. KEGG pathway enrichment analysis showed that various pathways were affected by LSD1 depletion in LSD1-silenced SW620 cells. The top 10 pathways included p53 signaling pathway, and pathways in cancer, ubiquitin-mediated proteolysis, axon guidance, aminoacyl-tRNA biosynthesis, small cell lung cancer, cell cycle, pyrimidine metabolism, B cell receptor signaling pathway, and chronic myeloid leukemia (Table 1).

**Figure 7:**
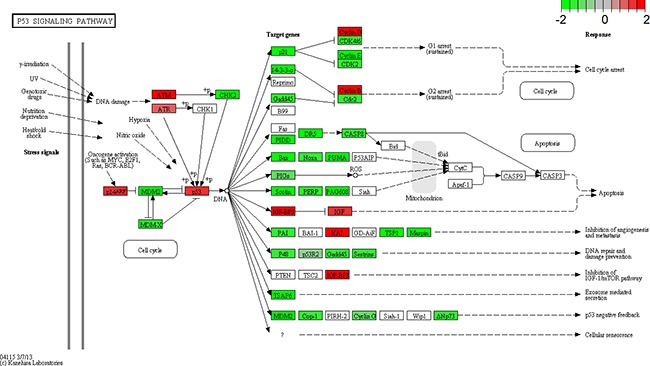
KEGG pathway analysis Gene expression for a subset of genes in the p53 signaling pathway is enhanced by lysine-specific demethylase1 (LSD1) depletion. p53 pathway is adapted from KEGG database. Upregulated genes are labeled in red, and downregulated genes are labeled in green.

Transcriptome analyses showed that p53 signaling pathway was the most frequent pathway affected by LSD1 depletion (Table 7, Figure 7). About 62 genes were involved in this pathway, of which 44 genes were differentially expressed in LSD1 depleted cells. Among these DEGs, six significantly DEGs were enriched in the p53 signal pathway (Figure 7, P < 0.05, FDR < 0.05). Surveys showed that LSD1 could not only suppress p53-mediated transcriptional upregulation, hold back apoptosis, but also conduce to human carcinogenesis in addition to chromatin modification [6].

**Table 7:** GO Pathway enrichment analysis of top10 probe sets

Gene set name/Pathway	Genes in overlap	P Value
hsa04115:p53 signaling pathway	**44**	8.86267304124769E-06
hsa05200:Pathways in cancer	**157**	3.33626652339401E-05
hsa04120:Ubiquitin mediated proteolysis	**73**	0.000120860811361763
hsa04360:Axon guidance	**69**	0.000164828739998662
hsa00970:Aminoacyl-tRNA biosynthesis	**27**	0.000507407177734314
hsa05222:Small cell lung cancer	**47**	0.000623475891805467
hsa04110:Cell cycle	**64**	0.00133161440702022
hsa00240:Pyrimidine metabolism	**49**	0.00449493011183359
hsa04662:B cell receptor signaling pathway	**40**	0.00535861984176549
hsa05220:Chronic myeloid leukemia	**40**	0.00535861984176549

### Validation of microarray data via RT-PCR and Western blot analysis

**Figure 8:**
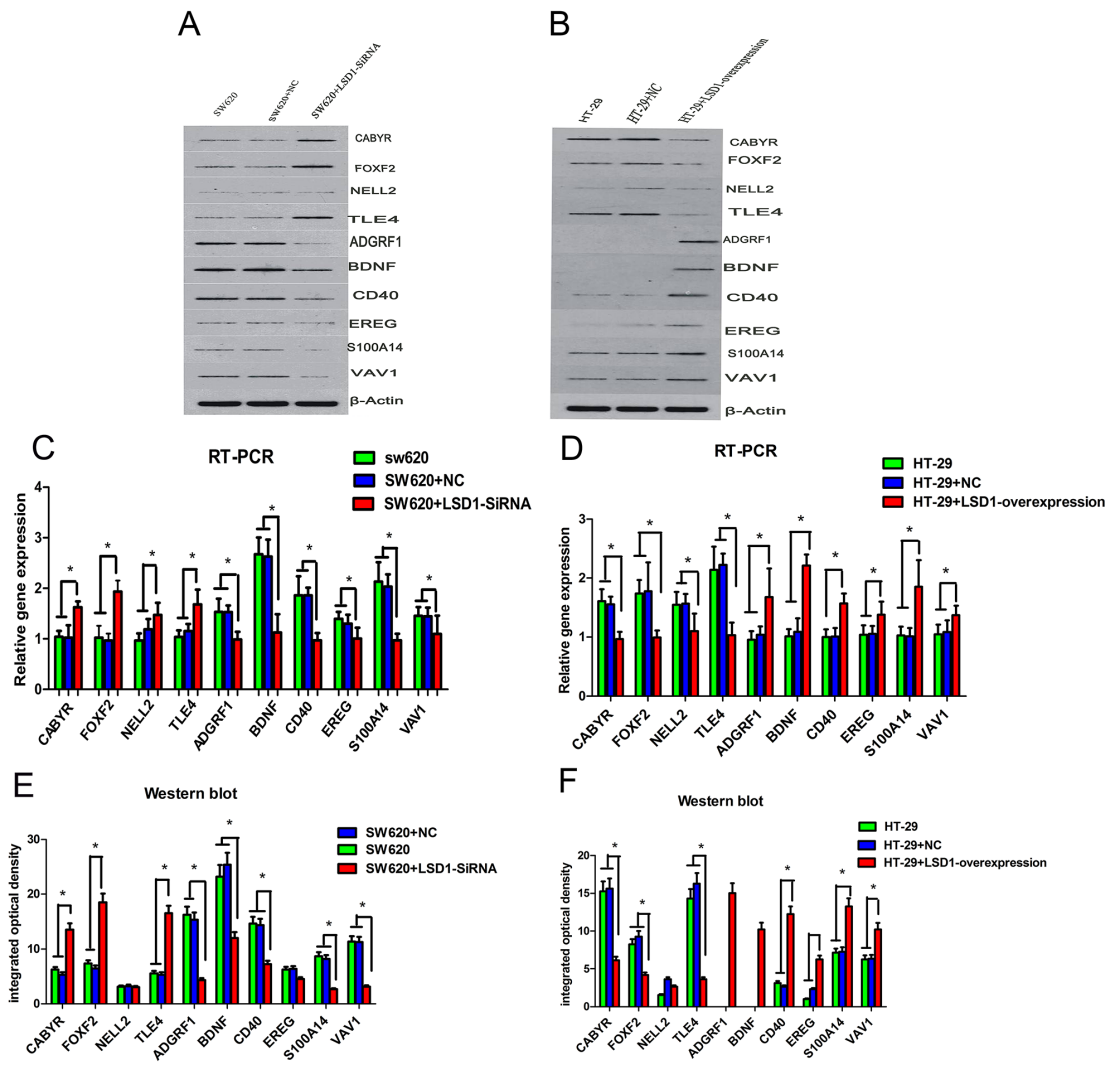
RT-PCR and western blot confirmation of microarray results for 10 DEGs LSD1-targets were detected in SW620 and HT-29 cell lines at both the RNA and protein levels. **A–B.** RT-PCR analysis of LSD1 in SW620 and HT-29 cells. **C.** CABYR, FOXF2, NELL2, and TLE4 show significantly high mRNA expression, whereas ADGRF1, BDNF,CD40, EREG, EREG, S100A14, and VAV1 show significantly low mRNA expression in SW620+LSD1-siRNA. **D.** ADGRF1, BDNF,CD40, EREG, S100A14, and VAV1 exhibit significantly high mRNA expression, whereas CABYR, FOXF2, NELL2, and TLE4 shows significantly low mRNA expression in HT- 29+ LSD1- overexpression. **E–F.** Western blot analysis of LSD1 in SW620 and HT-29 cells. The change trend of protein expression in HT-29+LSD1-overexpression, HT-29,and HT-29+NC cells is consistent with the mRNA expression of LSD1 in SW620+ LSD1- siRNA, SW620, and SW620+NC. * p <0.05.

**Figure 9:**
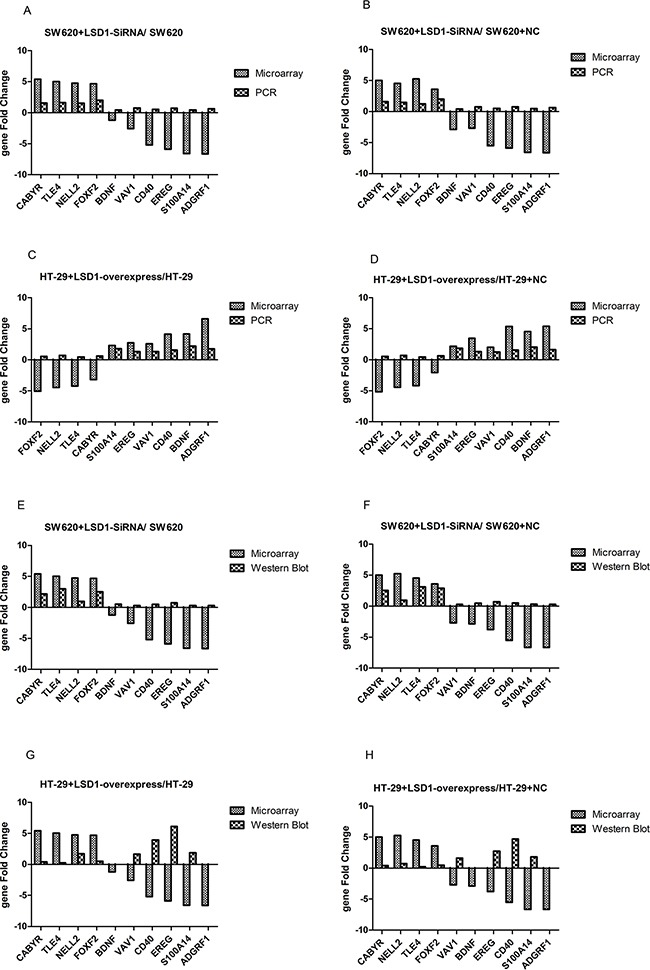
Comparison between gene chip data and PCR and Western blot results **A–B**. Comparison between microarray and RT-PCR analysis show that mRNA expression and DEGs change in the same direction in SW620+ LSD1- siRNA, SW620, and SW620+NC cells. **C–D**. mRNA expression and DEGs change in the same direction between RT-PCR and microarray in HT-29+LSD1-overexpression, HT-29,and HT-29+NC cells. **E–F**. Comparison between microarray and Western blot analysis show that protein expression and DEGs change in the same direction in SW620+ LSD1- siRNA, SW620, and SW620+NC cells. **G–H**. Comparison between microarray and Western blot analysis show that protein expression and DEGs change in the same direction in HT-29+LSD1-overexpression, HT-29,and HT-29+NC cells. The validation results indicate that microarray data correlated well with the PCR and Western blot results. Data are represented as means ± standard deviations.

Ten genes from set of D (Figure 1, Table 8) were chosen randomly for validation. Validation results indicated that PCR and Western blot results were concordant with the microarray analyses, and their fold changes were obvious at both RNA and protein levels (Figures 8 and 9).

**Table 8:** 10 DEGs were selected randomly to be validated from set of D (the intersection of III and γ) expressed as the ratio of experimental group and control group

GeneSymbo	Description	Genbank Accession	Fold ChangeC/A	Fold ChangeC/B	Fold ChangeG3/G1	Fold ChangeG3/G2
CABYR	Calcium Binding Tyrosine-(Y)-Phosphorylation Regulated	NM_153770	5.385	5.008	−3.183	−2.065
FOXF2	d ForkheadBox F2, eficiency promotes epithelial-mesenchymal transition and metastasis of basal-like breast cancer	NM_001452	4.674	3.582	−5.058	−5.140
NELL2	Neural EGFL Like 2	NM_001145108	4.748	5.247	−4.442	−4.448
TLE4	Transducin-Like Enhancer Of Split 4	NM_001282748	5.021	4.517	−4.234	−4.185
ADGRF1	Adhesion G Protein-C oupled Receptor F1	NM_153840	−6.644	−6.644	6.594	5.389
BDNF	Brain-Derived Neurotrophic Facto	NM_001143805	−2.207	−2.861	4.163	4.542
CD40	CD40 Molecule, TNF Receptor Superfamily Member 5	NM_001250	−5.190	−5.509	4.148	5.354
EREG	Epiregulin	NM_001432	−5.885	−3.778	2.729	3.462
S100A14	S100 Calcium Binding Protein A14	NM_020672	−6.600	−6.644	2.309	2.141
VAV1	Vav 1 Guanine Nucleotide Exchange Factor	NM_001258206	−2.564	−2.687	2.579	2.009

(log2|Fold change| ≥ 2, p <0.01).

set of D: DEGs intersection of SW620 cell and HT-29.

### Identification of CABYR and CDH1 as LSD1-target genes required for mechanism study of invasion and metastasis in colon cancer

**Figure 10:**
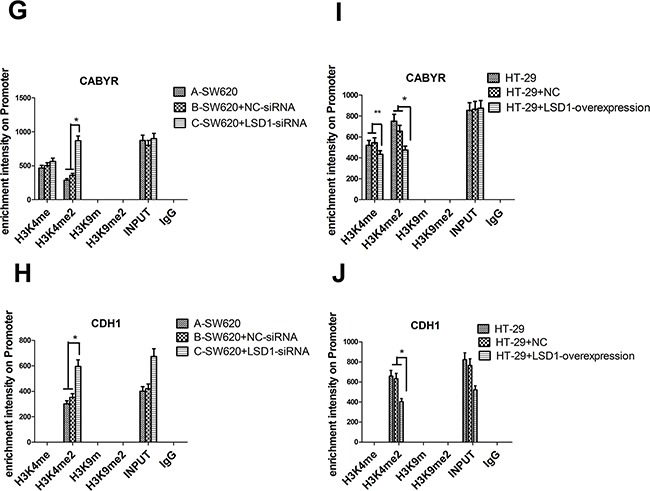
Effect of LSD1 on H3K4me1/2 and H3K9m1/2 methylations at the promoters of indicated genes **A**. Analysis of abundance of the LSD1-target gene promoter-bound LSD1 in SW620 and HT-29 cells, Knockdown of LSD1 by siRNA causes specific gene re-expression. Bands of CHIP and INPUT appeared in CABYR and CDH1. **C-D**. Silencing of LSD1 gene increases the expression of CABYR and CDH1 (*P <0.01,** P <0.05). **E-F**. LSD1- overexpression downregulates the expression of CABYR and CDH1(*P <0.01). **B**. ChIP analysis was used to determine the occupancy of H3K4me1/2 and H3K9m1/2 in the promoters of indicated genes. **G-H**. LSD1-depletion mediates the upregulation of CABYR and CDH1 expression through enhancing H3K4me2 occupations at LSD1-target gene promoter (*P <0.01). **I**. LSD1-overexpression mediates the downregulation of CABYR expression through decreasing H3K4me1/2 occupations at LSD1-target gene promoter region (*P <0.01,** P <0.05) and **J**. derepression of CDH1 through decreasing H3K4me2 (*P <0.01). RNA was extracted for CHIP-PCR analysis on expression of indicated genes. GAPDH is included as an internal control, and IgG is a negative control. Results are means ± standard deviations from three independent experiments. M = DNA marker.

ChIP was carried out to detect the occupancy of LSD1 at the target promoters in SW620 and HT-29cells and subsequently verify that the upregulation or downregulation of LSD1-target genes was a consequence of being a direct target of LSD1. Four significantly upregulated or downregulated LSD1-target genes, namely, CABYR, FOXF2, TLE4, and CDH1, were confirmed to be related to proliferation, apoptosis, tumorigenesis, invasion, and metastasis of cancer cells via bioinformatics technology (GO, Pathway studio) and literature search from DEG intersection of SW620 and HT-29 cells [26–31] (Figure 1, Table 3). LSD1 adjusts EMT through demethylation of CDH-1 gene involved in invasion and metastasis of cancer cells [32, 33]. Our findings suggested that knockdown of LSD1 by siRNA led to the increase in CABYR and CDH1 expression in SW620 cells. However, overexpression of LSD1 in HT-29 cells significantly decreased the expression of CABYR and CDH1 (Figures 10A, 10C-10F).

We examined the effect of LSD1- depletion and LSD1-overexpression on mono- and dimethylation of H3K4 and mono- and dimethylation of H3K9. The results suggested that LSD1- depletion mediated the upregulation of CABYR and CDH1 expression through enhancing the dimethylation of H3K4 at the LSD1-target genes promoter in SW620+ LSD1- siRNA cells. Similarly, LSD1-overexpression mediated the downregulation of CABYR expression through decreasing the mono- and dimethylation of H3K4 at LSD1-target gene promoter (*P<0.01) and downregulated CDH1expression through decreasing the dimethylation of H3K4 at LSD1-target gene promoter (Figure 10B, 10G-10J).

## DISCUSSION

The recent discovery of LSD1, which belongs to flavin-dependent amine oxidase family, is the first lysine-specific demethylase. It uncovered that histone methylation is reversible [34]. The balance of methylation and demethylation in epigenetic modification has an impact on gene expression and cellular activity. Many prior studies on LSD1 showed that aberrant histone lysine methylation in cancer is correlated with the repression of chromatin related to specific genes, and the repression of large chromosomal regions [7].

Up to now, only a few studies have involved in LSD1 in colon cancer. Studies involving knockdown of LSD1suggested that loss of LSD1 expression lessens the growth of cancer cells and their potential for migration and invasion [7]. In spite of this, it is uncertain that whether LSD1 has effects on proliferation, migration, and invasion in colon cancer. In the present study, we investigated the downstream LSD1- target genes in colon cancer cells via microarray gene expression profiling and ChIP promoter array to illustrate that epigenetic changes have relation with genetic changes in colon cancer. In addition, metastasis-related target genes were identified by bioinformatics technology.

LSD1 shows significantly high expression in colon cancer specimens. if LSD1 is inhibited, it could impair proliferation and invasiveness, and induce apoptosis of colon cancer cells *in vitro* [3, 35]. We constructed global gene expression profiles to analyze the LSD1-target gene expression in six experimental groups. Results showed that a significant difference existed between the experimental and control groups.

The regulatory network of LSD1 is determined by further functional analysis. KEGG pathway analysis using the KEGG database also confirmed that LSD1 could propose regulations on several cellular signaling pathways that embody the p53 signaling pathway which is crucially involved in cell apoptosis and metastasis, LSD1 also affected the IGF-1/mTOR pathways (Table 1 and Figure 6). LSD1 holds back the accumulation of dimethyl groups of p53, represses p53-mediated transcriptional upregulation, prevents apoptosis, and contributes to human tumorogenesis via a chromatin modification mechanism [6].

ChIP assay results suggested that LSD1 might be negative regulators for target genes CABYR and CDH1. Furthermore, the expression level of LSD1 is positively correlated with mono- and dimethylation of H3K4 at LSD1-target gene promoter region. Our previous studies indicated that LSD1 may facilitate the metastasis of colon cancer by decreasing the dimethylation level of H3K4 at the CDH1 promoter and by repressing E-cadherin transcription [4], which was consistent with the present results. No significant mono- and dimethylation of H3K9 were detected at the promoter regions of CABYR and CDH1. The present results are remarkable in that neither LSD1- depletion nor LSD1- overexpression alters the global levels of mono- and dimethylation of H3K9 or changes the activity of LSD1-target genes. LSD1 acts primarily as a histone demethylase that takes away the methyl groups from mono- and dimethylation of H3K4 to suppress gene expression [36]. Only in some prostate cancer cells can LSD1 bind to androgen receptor in a ligand-dependent manner to remove the methyl groups from mono- and dimethylation of H3K9 to activate gene expression [37].

A recent study showed that LSD1 inactivation induces a global increase of both mono- and dimethylation of H3K4 and mono- and dimethylation of H3K9 in Sox2-expressing cancer cells [38]. The role of mono- and dimethylation of H3K9 in transcriptional repression is probably a manifestation of a crucial positive role of mono- and dimethylation of H3K9 acetylation in signal-regulated transcription.

Additionally, lysine methylation can exist in three different states (mono-, di-, and trimethylation), which may cause additional regulatory complexity [39]. More studies clearly suggest that the loss of LSD1 strongly influences growth and changes gene expression profiles. LSD1 is a component of multiple transcriptional repressor complexes, so it is able to repress transcription broadly. Consequently, the genes or gene families whose expression is directly or indirectly affected by LSD1 can be determined by its ability we mentioned just now [40].

In summary, our data supply a molecular basis for the interaction of histone demethylation in chromatin remodeling, these data also indicate that LSD1 may inhibit the p53 and IGF-1/mTOR signaling pathways, prevent apoptosis and contribute to human carcinogenesis through enhancing dimethylation of H3K4 at LSD1-target gene promoter region. In this study, we assessed these possible LSD1-targets, including CABYR and CDH1, which could be the newly identified target genes by comparing the effects of LSD1 in the two colon cancer cells. Epigenetic control of gene regulation has boundless potential and is a rapidly developing field. The discovery of epigenetic markers implicates that future work should be addressed the mechanism on the definition of potential targets for epigenetic therapy.

## MATERIALS AND METHODS

### Human colon carcinoma cell lines and cell culture

Colon cancer cell lines used in this study were purchased from American Type Culture Collection (Sigma-Aldrich Corp, St Louis, MO, USA). HT-29 cells were cultured with RPMI-1640 medium (Sigma- Aldrich), and SW620 cells were cultured with L-15 medium (Sigma- Aldrich). All media were supplemented with 10% fetal bovine serum and 1% antibiotic/antimycotic solution (Biowest, Nuaille, France). All of the cell lines were grown in 5% CO2 at 37°C in incubators with 100% humidity.

Considering SW620 cell line was more aggressive, and HT-29 was less aggressive than other colon cancer cell lines [3], both of them were selected in the experiment. Colon cancer cell lines SW620 and HT29 were cultivated *in vitro and* divided into six groups according to different treated factors, as shown in Table 9.

**Table 9:** Grouping of the experiment according to different treated factors

group	Cell group
**A**	SW620
**B**	SW620+negative control (NC)
**C**	SW620+ LSD1- siRNA (transfection of LSD1 siRNA)
**G1**	HT29
**G2**	HT29+ negative control (NC)
**G3**	HT-29+LSD1-overexpression (overexpression of LSD1)

### siRNA and LSD1- overexpression vector transfection

The cells were seeded with 5×10^4^ cells in 24-well plates and then incubated for 2–4 days in standard medium in the presence of 10-20 nmol/L siRNA directed against LSD1. The siRNA LSD1 sequences used in this study were as follows: siLSD#1 (sense: 50-GCCACCCAGAGAUAUUACUTT-30, anti-sense:50-AGUAAUAUCU CUGGGUGGCTT-30); siLSD#2 (sense: 50-CCGGAUGACUUCUCAAGAATT-30, anti-sense: 50-UUCUUGAGAAGUCAUCCGGTT-30); siLSD#3 (sense: 50-CCACGAGUCAAAC CUUUAUTT-30, anti-sense: 50-UUCUUGAGAAGUCAUCCGGTT-30), siLSD#3 (sense: 50-CCACGAGUCAAAC CUUUAUT T-30, anti-sense: 50-AUAAAGGUUUGAC UCGUGGT T-30), or control siRNA (sense: 50-UUCUCCGAACGUGUC ACGUTT-30, anti-sense:50-ACGUGACACGUUCGGAGA ATT-30). The cells were transfected using Lipofectamine 2000 (Invitrogen) according to manufacturer's instructions [3]. Overexpression plasmid LSD1 (5 μg) was transfected into HT-29 cell line in six-well plates (1 μg /mL) using the Lipofectamine reagent (Invitrogen) according to the manufacturer's instructions [7].

### Microarray analysis

The small RNA fragments were enriched with NanoSep 100K (Pall Corporation, USA) and desalted by flowing through Vivaspin 500 3k (Sartorius Stedim Biotech) from 2.5 μg of total RNA. The fluorescent targets were prepared using miRNA ULSTM Labeling Kit (Kreatech Diagnostics, Netherlands). The labeled fluorescent targets were hybridized to prehybridized mouse miRNA OneArray v5 (Phalanx Biotech Group, Hsinchu, Taiwan). Nonspecific binding targets were washed away after 16 h hybridization at 37°C, Slides were dried via centrifugation and scanned using an Axon 4000B scanner(Molecular Devices, Sunnyvale, CA, USA). The Cy5 fluorescent intensities of each spot were analyzed using GenePix 4.1 software (Molecular Devices).

The signal intensity of each spot was processed by R program. The median value of the repeating spots was selected for analysis. We filtered out spots with flag that was <0 within all arrays. Spots that passed the criteria were normalized through invariant set normalization method. Normalized spot intensities were transformed to gene expression log2 ratios in the pairwise t-test between the control and treatment groups. The spots with |log2 ratio| ≥0.8 and P-value <0.05 were tested for further analysis. Target genes and functions prediction for selected differential miRNAs were analyzed by NTU miRSystem website (NTU, Taipei, Taiwan).

### Real-time PCR

The total RNA was extracted using Trizol reagent (Invitrogen, Carlsbad, CA, USA). cDNA synthesis was performed using the RevertAid First Strand cDNA Synthesis Kit (Fermentas, Vilnius, Lithuania). Gene expression of LSD1 was monitored via RT-PCR using Assays-on- Demand (Applied Biosystems, Alameda, CA, USA). The expression values were normalized to the geometric mean of GAPDH. The primers used to amplify cDNA are shown in Table 10.

### Western blot

Protein lysates were extracted from the cells and blotted as described previously [41]. The membranes were incubated for 1–2 h using the following antibodies and dilutions: TLE4, ADGRF1, BDNF, CD40, EREG, and S100A14: 1:500; FOXF2, NELL2, and, VAV: 1:1000; β-Acti, 1:800; and secondary antibodies and dilutions: 1:4000.

### ChIP

ChIP was performed with Chromatin Immunoprecipitation Kits (Active Motif, Carlsbad, CA, USA) according to manufacturer's instructions. Briefly, cells were fixed after various treatments with TPA. Histone modifications H3K4me2/ H3K4me, H3K9m/H3K9me, and LSD1 occupancy at LSD1-target promoter were examined [42].

Results were quantified with RT-PCR. Signals were normalized to isotype control IgG. Six pairs of primers, which covered 1 kb range of LSD1-target promoter region, were employed. Sequences of primers used for ChIP assays are shown in Table 11.

**Table 11:** Sequences of primers used for ChIP assays

Gene	Forward primerReverse primer	Product Size (bp)
Homo-CABYR-FHomo-CABYR-R	5-CAGGAGCAATGTGTGGAATG-35-GGCGAGGGAGACTTATTTCC-3	204
Homo-FOXF2-FHomo-FOXF2-R	5-GGAAGCAAATGCAACCCTAA-35-CCAAAGGTAACGTGCCAATC-3	218
Homo-CDH1-FHomo-CDH1-R	5-AGTCCCACAACAGCATAGGG-35-TTCTGAACTCAGGCGATCCT-3	204
Homo-TLE4-FHomo-TLE4-R	5-AAAGAGGCGCAGTCTTGGTAT-35-TGAAGATCCGGGAATCAGAG-3	**177**
Homo-BDNF -FHomo-BDNF -R	5-TGGGAAGCCATAACCCATTA-35-AGCCCAACAACTTTCCCTTT-3	162
Homo-GAPDH-FHomo-GAPDH-R	5-TACTAGCGGTTTTACGGGCG-35-TCGAACAGGAGGAGCAGAGAGCGA-3	**166**

### Statistical analysis

Statistical analysis was performed using SPSS software (version 17.0; SPSS Inc, Chicago, IL, USA). Student's t-test, Benjamini-Hochberg, and pair wise t-test were performed to analyze differences between groups. Pearson chi-square test was used to investigate the correlation between differentially expressed gene (DEG) and GO annotation lists. Microarray analysis was performed with GCOS software of Affymetrix, GeneSpring, and Partek of Agilent. P Values<0.05 were considered significant.
